# Improving Outcomes in Mental Health (IOMH)—an Australian longitudinal clinical study of families with children with neurodevelopmental problems: cohort profile

**DOI:** 10.1136/bmjopen-2024-091676

**Published:** 2025-03-13

**Authors:** Dana E Galligan, Leanne Payne, Daniel P Sullivan, Madhura Bhadravathi Lokeshappa, Laura Ziser, Lorelle Nunn, Leanne M Wallace, Isabella Andersen, Sophie Howarth, Akina Kato, Mohan Karunanithi, Cassandra Mingin, Sally O’Scanaill, Nisreen Aouira, Ayu Paramecwari, Matthew R Sanders, Vanessa E Cobham, Naomi R Wray, Anjali K Henders, Enda M Byrne, Honey Heussler, Christel M Middeldorp

**Affiliations:** 1 Child Health Research Centre, The University of Queensland, Brisbane, Queensland, Australia; 2 Child and Youth Mental Health Service, Children’s Health Queensland Hospital and Health Service, Brisbane, Queensland, Australia; 3 Thompson Institute, University of the Sunshine Coast, Sippy Downs, Queensland, Australia; 4 Department of Psychology, The Prince Charles Hospital, Chermside, Queensland, Australia; 5 Institute for Molecular Bioscience, The University of Queensland, Brisbane, Queensland, Australia; 6 The University of Queensland Parenting and Family Support Centre, Brisbane, Queensland, Australia; 7 School of Psychology, The University of Queensland, Brisbane, Queensland, Australia; 8 Queensland Brain Institute, University of Queensland, Brisbane, Queensland, Australia; 9 Child Development Program, Children’s Health Queensland Hospital and Health Service, Brisbane, Queensland, Australia; 10 Department of Child and Adolescent Psychiatry and Psychology, Amsterdam UMC, Amsterdam Reproduction and Development research Institute, Amsterdam Public Health Research Institute, Amsterdam, The Netherlands; 11 Arkin Mental Health Care, Amsterdam, Amsterdam; 12 Levvel, Academic Center for Child and Adolescent Psychiatry, Amsterdam, The Netherlands

**Keywords:** Child & adolescent psychiatry, Depression & mood disorders, Anxiety disorders

## Abstract

**Abstract:**

**Purpose:**

Children with neurodevelopmental disorders (NDDs) such as autism spectrum disorder (ASD) and attention-deficit hyperactivity disorder (ADHD) face a range of challenges which impact their daily functioning and that of their family. NDDs are often associated with significant mental health problems which can influence the course. The Improving Outcomes in Mental Health cohort described in this article aims to investigate the risk factors for the persistence and severity of mental health problems in children with NDDs.

**Participants:**

A total of 1084 families (primary caregivers and children) were recruited from the Child Development Program at the Children’s Health Queensland Hospital and Health Service in Brisbane, Australia. 1471 caregivers (female n=1036) participated in the study, which included 382 families with 2 or more caregivers participating. The children were predominantly male (71%), with the average age of all children 5.6 years.

**Findings to date:**

The most prevalent child clinical diagnoses were ASD and ADHD, with half of children receiving more than one diagnosis. Caregiver reports indicated that children were experiencing clinical levels of depression (30.8%) and anxiety (27.6%). Approximately 39% of caregivers scored in the subclinical or clinical range for at least one Diagnostic and Statistical Manual of Mental Disorders measure, the majority reporting depressive problems.

**Future plans:**

Future plans for this data set include analysis of environmental variables such as family structure, income, school achievements and leisure activities as risk factors for the persistence of mental health problems in children with NDDs. Genetic data will be used to provide insights into the heritability of mental illness and improve prediction.

**STRENGTHS AND LIMITATIONS OF THIS STUDY**

- Clinical patients were tracked at up to four timepoints allowing the trajectory of mental disorders to be measured along with assessing treatment efficacy.
- Recruitment included participation of more than one caregiver, allowing a rounded picture of the child’s symptoms and family history.
- A rich variety of data, including clinical, demographic, behavioural and biological, was collected.
- Data collection concluded at the end of 2023 due to funding. Although all data were retained, this resulted in some participants not being invited to participate in later timepoints and increased the rates of attrition.

## Introduction

 Neurodevelopmental disorders (NDDs) encompass a broad range of mental disorders which are characterised by an onset in early childhood and include intellectual developmental disorders, communication disorders, autism spectrum disorders (ASDs), attention-deficit/hyperactivity disorders (ADHDs), specific learning disorders and motor disorders.^1^ Presentation is heterogeneous and symptoms can include both behavioural, social, executive functioning and/or cognitive problems.

The two most common NDDs are ADHD and ASD.^2^ Globally, ADHD affects 3.4% of children and adolescents^3^ and is characterised by hyperactivity, impulsivity and/or inattention, with hyperactive/disruptive symptoms typically mostly seen in early childhood.^1 4^ ADHD diagnosis is classified into three subtypes: predominantly hyperactive/impulsive, predominantly inattentive or combined presentation meeting criteria for both inattention and hyperactivity-impulsivity. ASD is characterised by deficits in social communication and interaction skills, and repetitive behaviours, interests or activities, resulting in impairments in everyday functioning.^1^ The prevalence of ASD is 1 in 54, with boys 4.3 times more likely to experience ASD than girls at age 8.^5^

The course of ADHD and ASD varies. Around 70% of ADHD cases persist into adulthood.^6 7^ The trajectory of ASD is generally considered chronic, persisting into adulthood; however, cases can remit with symptoms undetectable.^8 9^ Irrespective of symptom persistence into adulthood, those who experience NDDs in childhood face poorer educational and employment outcomes, greater social isolation and reduced quality of life.^10^,^13^ Understanding the course of NDDs is important to improving these outcomes.

One of the factors influencing the course are comorbidities, which are common in people with NDDs, with approximately 30% experiencing more than one NDD, and 58% also experiencing an additional non-NDD psychiatric disorder.^14 15^ Although considered distinct disorders, ADHD and ASD symptoms can become entangled and diagnosis of one and/or the other can become difficult.^1 16^ Children with co-occurring ASD and ADHD are at a higher risk of other co-occurring psychiatric conditions, including depression and anxiety.^17^ Anxiety affects approximately 40% of children and adolescents with ASD across all age groups,^18 19^ and can contribute to increased levels of social communication impairment.^20^ Depression co-occurring with ASD affects 11% of children and adolescents, with boys experiencing more symptoms in early adolescence than girls; this sex difference disappears by age 21.^21^ Young people with ADHD and depression experience more severe depression symptoms than those without ADHD.^21^ Another known factor influencing the course is family history due to both genetics and environmental influences, and the interaction between both.^22^ Twin studies have shown heritability ranging from 30% to 50% for both depression and anxiety,^23 24^ whereas neurodevelopmental disorders are generally higher, with ADHD 70–80%^25^ and ASD 64–91%^26^

Still, knowledge on factors influencing course is limited and hence it is not possible to stratify children into groups of high and low risk for an unfavourable course and provide targeted treatment aimed at optimising the course of symptoms and avoiding comorbidity. Mental disorders are complex and multifactorial in nature and cannot be attributed to a single cause. It is likely that this also applies to the course of mental disorders. To improve the identification of children at high risk for an unfavourable course, we collected an extensive range of environmental and biological data. Factors such as lower childhood socio-economic status,^27^ parental age,^28 29^ parental mental health symptoms^30^ and leisure activities^31^,^33^ have been associated with NDDs.

Biological data were also collected to help improve understanding of the underlying biology of NDDs and their association with comorbidities and behavioural measures. NDDs are highly heritable, and understanding the genetic basis will provide insight into their course. ADHD and ASD are known to be highly polygenic, with many genetic variants contributing to risk.^34^,^36^ Combining the effect of many genetic variants, a polygenic risk score (PRS) can be calculated to quantify a person’s individual risk. A recent study revealed genetic overlap between ASD, ADHD and depression.^35^ PRSs have been shown to have a predictive capacity regarding the course of mental disorders such as depression.^37^

Microbiome and metabolomic data were also collected to investigate their role in the progression of mental disorders. There has been recent interest surrounding mental disorders and the gut-microbiota-brain axis. The gut microbiota is extremely dense, containing millions of microorganisms,^38^ and can act to regulate nervous system function.^39^ The gut-microbiota-brain axis acts as a pathway allowing bidirectional communication between gut microorganisms and the brain.^40^ The gut microbiome has been associated with ASD,^41^ ADHD,^42 43^ anxiety and depression.^44^ Metabolites are the by-products of various metabolic functions in our bodies and lie downstream of genomic and microbiome interactions. Analysis of metabolites in human biofluids such as urine can provide sensitive phenotype measures.^45^

This rich data set will aid in understanding the complexity of NDDs.

## Cohort description

### Participants

Families (primary caregivers and children) in which a child, aged between 2 and 18 years, had been referred to one of the Child Development Services at the Children’s Health Queensland Hospital and Health Service (CHQHHS) for an assessment were contacted for recruitment between 2018 and 2023. Child Development Services are multidisciplinary, assessing and offering interventions for children with developmental concerns. The service comprises 12 clinics in the greater Brisbane region. The study started with a pilot with participants recruited from one clinic located at the Queensland Children’s Hospital (QCH). Recruitment expanded after 14 months to include two additional CHQHHS clinic locations (Northlakes and Coorparoo). Further clinic expansion was delayed due to COVID-19 but ultimately included recruitment from all 12 clinic locations.

All English-speaking primary caregivers (parents, stepparents, grandparents, aunts/uncles) of the referred children were invited to participate. At least one legal guardian was required for participation, and consent was obtained for participation and access to the child’s clinical data. Children in the care of the Department of Child Safety were excluded from participation due to difficulties regarding obtaining consent from a legal guardian. A total of 1084 families (1471 caregivers and 1084 children) were recruited. All caregivers in each family were encouraged to participate, allowing each caregiver to provide information on their own symptoms and family history as well as their child’s mental health symptoms. Hence, for some children there are two parents or carers providing information on them. Caregivers within each family were assigned a label to distinguish each family member (P1=parent 1, P2=parent 2, Other=caregiver who is not a parent, eg, a grandparent). These labels remained with the participant for the entirety of the study.

### Data collection

The IOMH study participants completed up to four assessments. The first assessment was part of routine clinical practice. Families who were referred to a child development service were requested to complete the initial assessments before their first appointment. The results were stored in their file on the Queensland Health integrated electronic Medical Record (ieMR) to support the diagnostic assessment and formed the baseline (T1) survey results. Consent was obtained for use of these data for research, as well as the information on diagnoses. Families were also approached and asked for biological samples specifically for the purpose of research.

As the time to complete the diagnostic assessment varied depending on the number of needed assessments, it was decided that families would be contacted to complete the timepoint two (T2) survey 6 months after a formal diagnosis was received from the clinic. This allowed for the follow up to be completed at a similar timepoint in each patient’s journey as treatment mostly starts only after the diagnosis is made. If no diagnosis had been made nine months after the first assessment, the caregivers were then invited to complete T2. A randomised controlled trial (RCT) was also embedded in the longitudinal study for families in which parents scored in the sub-clinical or clinical range in any of the Adult Self Report scales at T1. The RCT aimed to investigate if participation in an enhanced parenting program (Enhanced Stepping Stones Triple P) addressing parental mental health symptoms provided additional benefits to child mental health over and above a standard parenting program (Stepping Stones Triple P) . Parents who participated in the trial were asked to complete the T2 questionnaire on completion of the trial intervention.

All families were invited to complete the timepoint 3 (T3) questionnaire 6 months after the completion of T2 or 6 months after the due date for T2 if T2 was not completed. Timepoint 4 (T4) occurred either 12 months after the completion of T3 or 12 months after the T3 due date if participants did not complete T3. Additionally, if both T2 and T3 were not completed, T4 was scheduled 18 months after the T2 due date. Biological samples were collected at T1 and T2 or at later timepoints if they had not been collected at previous timepoints.

Recruitment into the study concluded in March 2023. Data collection for T2–T4 remained open for participants to complete until November 2023. As a consequence, some families did not have the opportunity to participate in the follow-up time points.

### Participant and patient involvement

A parent of patient group was consulted for feedback surrounding the survey. Feedback from participants regarding biological collections was taken into consideration and implemented when practicable. Throughout the study, participants were provided regular updates through email newsletters. Research results will be shared with participants by email.

**Table 1:** Data collection

Collection schedule	T1	T2	T3	T4
	Baseline	6 months	12 months	24 months
Questionnaire data	✔	✔	✔	✔
Biological samples	✔	✔	✓	
Questionnaire data				
Parent and child demographic information				
Parent name, date of birth, relationship to child				
Child name, child date of birth				
Aboriginal and/or Torres Strait Islander Status				
Ancestral group				
Language spoken at home				
Postcode				
Family information				
Family type				
Does child live at another household?				
How many nights per week?				
Who are they living with when not with parent?				
Others living in the household?				
Number of persons living in the household				
Parent information				
Highest level of education				
Current work status				
Household income				
Psychological disorders				
Family history of mental illness				
Medical conditions				
Medication use				
Concerns about the child’s health				
Participation in parenting or child development programmes				
Mental health symptoms—Adult Self Report				
Child information				
Medical conditions				
Medication use				
Daytime activities				
Extracurricular activities				
Screen time				
Academic achievement				
Mental health symptoms—Child Behavior Checklist				
Autism symptoms—Social Responsiveness Scale—Short Form				
COVID-19 information				
Have you, your child or family:				
Fallen ill, required hospitalisation, self-isolated or passed away?				
Biological samples				
Urine				
Stool				
Australian Eating Survey				
Australian Child and Adolescent Eating Survey				
DNA (blood, saliva or buccal swab)				

Collection Schedule: Questionnaires were collected at Baseline (T1), then 6 months (T2), 12 months (T3) and 24 months (T4) from clinical diagnosis. Biological samples were collected at T1 and T2. If this did not occur, for example due to COVID closures, they were collected at T3. Families in which one or more parents scored clinical or sub-clinical in the T1 Baseline assessment were invited to participate in the randomised controlled trial.

### Retention

Given the longitudinal nature of the study, attention was given to retaining participants for the entirety of the study. In addition to contacting participants directly by phone and text message for questionnaire and sample completion, families were sent newsletters at each timepoint updating on the study progress, and handwritten birthday cards were sent to participating children. Families were sent a $25 gift card on completion of T2, T3 and T4 as reimbursement for their time.

### Data collected

#### Questionnaire

At each timepoint, a questionnaire which included information such as demographics, family structure, work, education, health and extracurricular activities was completed by parents (table 1). The questionnaire also addressed a broad range of psychiatric problems to account for comorbidities, and the homotypic and heterotypic presentation of symptoms over time. Given the parent-offspring associations in psychiatric problems, and in the course of the problems, parents were also asked to report on their own problems. Mental health symptoms in parents and children were assessed with the appropriate age versions of the Achenbach System of Empirically Based Assessment. The Child Behavior Checklist (CBCL)^46^ was completed by caregivers for children aged 6–18 years, and the Preschool CBCL was completed for children aged 2–5 years. The CBCL and CBCL Preschool version consists of 113 and 100 Likert-like questions, respectively, assessing behaviour and emotion in children. The ASR^47^ was completed by caregivers and contains 126-item Likert-like questions that assess behaviour and emotion in adults. The Social Responsiveness Scale—Short Form (SRS)^48^ was completed by caregivers to screen children aged 6–18 years for autism traits, specifically reciprocal social behaviour. The SRS contains 16 Likert-like questions, with total scores ranging from 0 to 48. Questions regarding the impact of COVID-19 were added in April 2020 to surveys at all timepoints and remained in the survey until the end of 2022. After the initial survey development, a parents/carers of patients group was consulted for feedback surrounding the survey. From this feedback, questions regarding positive experiences and siblings were incorporated into the survey.

#### Biological sample collection

Biological sample collection was refined and adapted over the duration of the study due to funding, COVID-19 restrictions, participant feedback and uptake. Caregivers and children consenting to data use were asked to provide blood, urine and stool samples at T1 and T2. For those participants not attending the QCH, a trained phlebotomist travelled to their homes to collect a blood sample, along with the frozen urine, and stool samples for analysis. Recruitment expanded to 12 clinics across the greater Brisbane region; however, funding was not sufficient to support home visits on this larger scale, and as a result only samples that the families could mail back (saliva, buccal swabs and stool) were collected. COVID-19 lockdowns led to home visits and all sample collection being suspended from March 2020. Sample collection recommenced in June 2020 with the introduction of saliva samples as a less invasive option. Saliva samples were subsequently discontinued (September 2020) as children found it difficult to provide the required sample volume. As a result, collection changed to buccal swab. Stool was then reintroduced. Blood samples were still taken at T2 for the families who provided blood at T1 and were then discontinued. There was low uptake of stool swabs at T2, and as a result, from February 2022, the collection of stool swabs at T2 was dropped. The final sample protocol comprised: T1—stool and buccal swab, T2—buccal swab and T3—buccal swab only if no DNA sample had been collected at prior timepoints.

**Table 2:** Caregiver age at baseline, demographic information and Adult Self Report scores

			n	Mean (SD)
**Age**				
Parent/guardian			1466	36.3 (7.1)
Female			1031	35.4 (6.9)
Male			435	38.4 (6.9)
Other family member female			5	55.8 (14.1)
Parent ethnicity			**n**	**%**
Caucasian			1060	(72.4)
Asian			176	(12.0)
Pacific Islander			84	(5.8)
Indigenous			64	(4.4)
Other			79	(5.4)
Parent employment status			**n**	**%**
Employed full time (35+ hours per week)			557	(38.1)
Employed part-time or casual			431	(29.5)
Not in paid employment			403	(27.5)
Student			69	(4.7)
Other			3	(0.2)
Parent level of education			**n**	**%**
Postgraduate study			175	(12.0)
University degree			313	(21.4)
Vocational college, certificate or diploma, trade			530	(36.2)
High school			416	(28.4)
Other			29	(2.0)
Family satus			**n**	**%**
Original family (both biological or adoptive parents present)			701	(64.7)
Sole parent family, after divorce or separation			239	(22.1)
Stepfamily (two parents, one being a stepparent)			64	(5.9)
Sole parent family, other parent never involved or deceased			62	(5.7)
Other			17	(1.6)
Household income ($)			**n**	**%**
1–49 999			281	(25.9)
50 000–99 999			331	(30.6)
100 000–149 999			204	(18.8)
150 000 or more			111	(10.2)
Prefer not to say			67	(6.2)
Don't know			70	(6.5)
No income			19	(1.8)
Parent mental health diagnosis			**n**	**%**
Yes			524	(35.8)
No			912	(62.3)
Prefer not to say			27	(1.8)
Family history of psychological diagnosis family history			**n**	**%**
Yes			774	(52.9)
No			634	(43.3)
Prefer not to say			55	(3.8)
**PrimarycaregiverAdult Self Reportscores**	**Mean (SD)**	**Clinical**	**Subclinical**	**Normal**
		**n (%)**	**n (%)**	**n (%)**
Adult Self Report				
Depression	6.2 (5.1)	161 (11.0)	207 (14.2)	1094 (74.8)
Anxiety	5.2 (3.2)	108 (7.4)	120 (8.2)	1234 (84.4)
Somatic	2.6 (3.0)	106 (7.3)	141 (9.6)	1215 (83.1)
Avoidant	3.7 (3.0)	169 (11.6)	101 (6.9)	1192 (81.5)
Attention-deficit hyperactivity disorder	5.8 (4.9)	119 (8.1)	98 (6.7)	1245 (85.2)
Antisocial	2.9 (3.2)	56 (3.8)	54 (3.7)	1352 (92.5)

Demographic statistics are reported for each primary caregiver as an individual, except family status and household income which are reported for each family. Household income is reported in Australian dollars.

Participants who provided stool samples also completed the adult Australian Eating Survey (AES)^49^ or the Australian Child and Adolescent Eating Survey (ACAES).^50^ Both the AES and ACAES contain 120 Likert-like questions to assess dietary behaviour (consumption of fruit, vegetable, dairy, snack foods, beverages, bread) and sedentary activities over the previous 6 months. The Bristol Stool Form Scale^51^ was completed by caregivers to categorise stools into seven categories using everyday descriptions and pictures. Both surveys were completed to assess diet and stool consistency which have both been associated with microbiota composition^52 53^

### Findings to date

**Figure 1:**
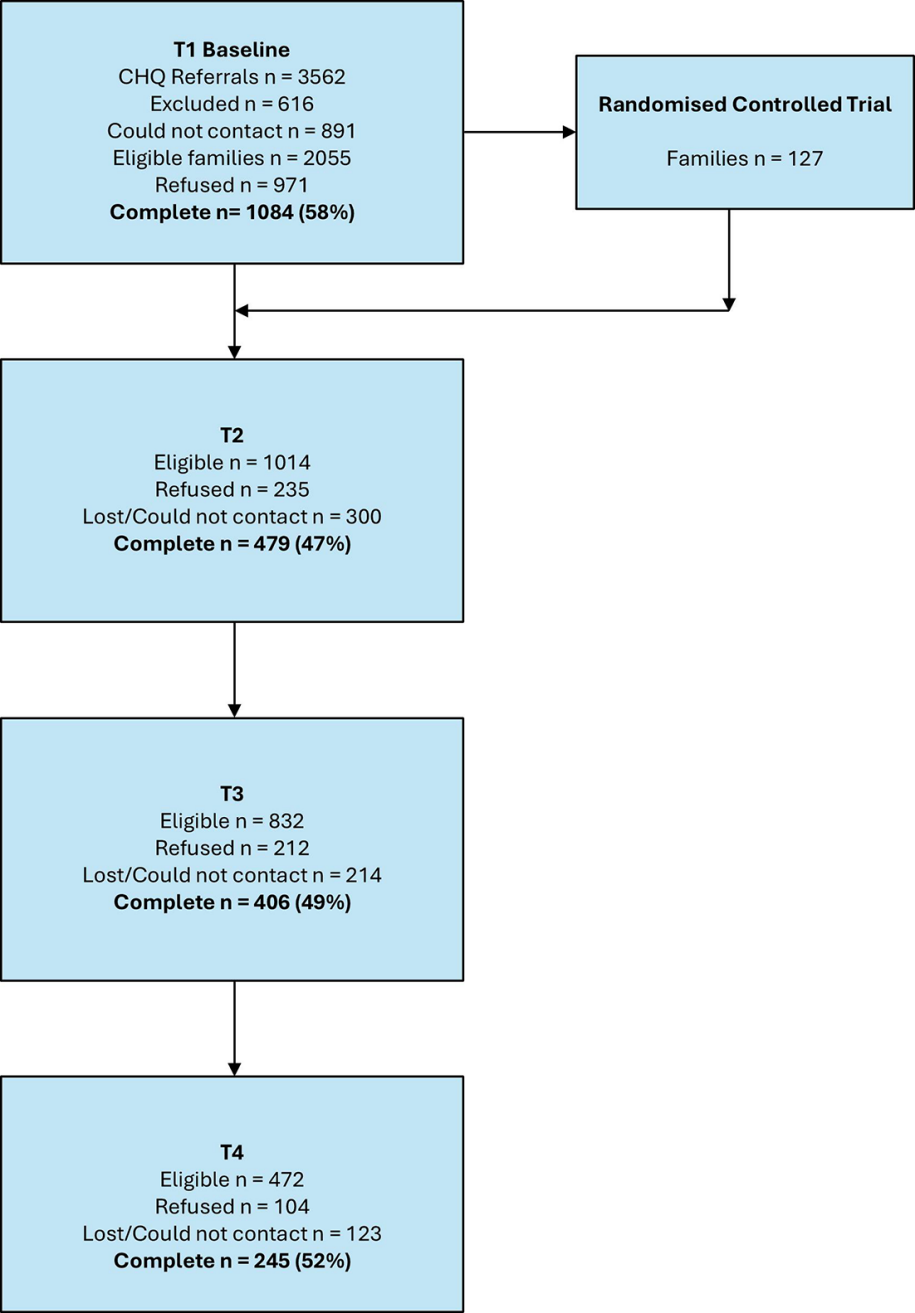
Family completion rates at each timepoint. Families were excluded from participation if the caregiver was not the legal guardian or were non-English speakers, or if the child was less than 2 years of age. After completion of T1, participants were able to refuse participation at a timepoint and then re-enter the study at a later timepoint. CHQ, Queensland Children’s Hospital.

**Figure 2:**
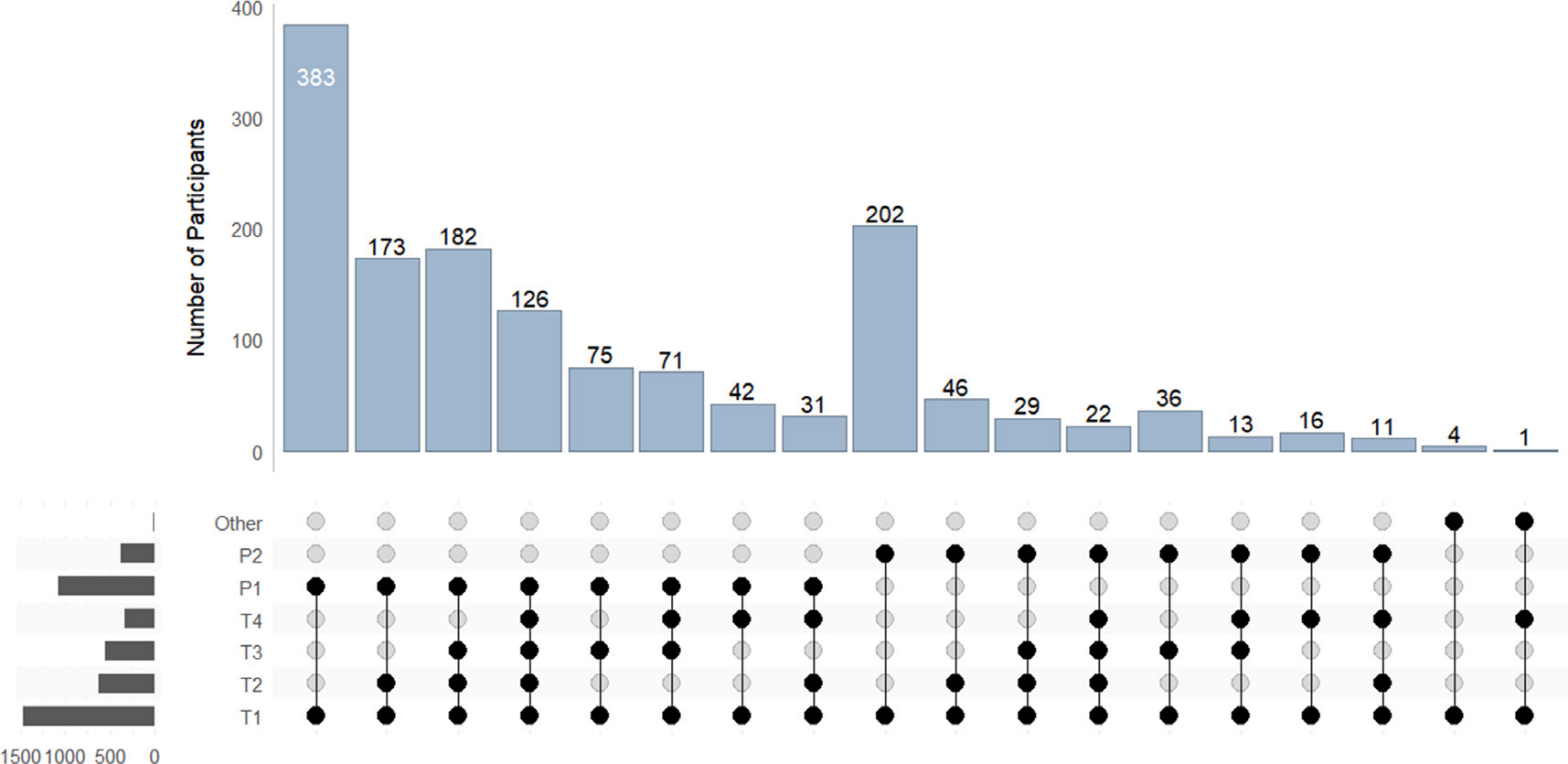
Survey completion by caregivers. Upset plot displays the completion of surveys at each timepoint by caregivers represented by dots. Each caregiver in a family is uniquely identified by either: P1, parent 1; P2, parent 2; other, caregiver who is not a parent, for example, a grandparent.

3562 families were referred to the Child Development Program during the recruitment period, with 1084 families eligible and consenting to participate in the study. Figure 1 shows the flow of families through each timepoint. Of the 1084 families that completed T1, 148 completed all four timepoint surveys. Figure 2 displays the timepoint completion for all caregivers.

### Demographics

The majority of families taking part consisted of one caregiver completing the questionnaire (n=701). The remaining families (n=382) had two caregivers taking part, resulting in two questionnaires completed for those children. The majority of caregivers enrolled were parents or guardians (n=1466), 70% of which were female. The average age was 36.3 years (SD=7.1) for parents or guardians, and 55.8 years (SD=14.1) for other primary caregivers (table 2) and 5.6 years (SD=2.7) for children (table 3).

**Table 3:** Child age at baseline, diagnosis, Child Behavior Checklist and Social Responsiveness Scale—Short Form scores

			n	Mean (SD)
**Age**				
Child			1084	5.6 (2.8)
Female			313	6.0 (2.9)
Male			771	5.4 (2.7)
Diagnosis			**n**	**%**
Autism spectrum disorder			301	27.8
Hyperkinetic disorders/attention-deficit hyperactivity disorder			292	26.9
Developmental delay (includes intellectual impairment)			225	20.8
Speech and/or language delay			224	20.7
Other mental disorders			379	35.0
Does not meet diagnostic criteria			216	19.9
Not able to make a diagnosis			56	5.2
Child age (years), n=1084			**Male n**	**Female n**
2–5			458	150
6–18			313	163
**ChildBehavior Checklist1–5**	**Mean (SD)**	**Clinical**	**Subclinical**	**Normal**
		**n (%)**	**n (%)**	**n (%)**
All caregivers				
Affective	5.1 (3.6)	260 (32.1)	61 (7.5)	490 (60.4)
Anxiety	5.9 (4.2)	188 (23.2)	50 (6.2)	573 (70.7)
Pervasive development	9.7 (5.2)	450 (55.5)	121 (14.9)	240 (29.6)
attention-deficit hyperactivity disorder	7.7 (3.2)	195 (24.0)	91 (11.2)	525 (64.7)
Oppositional defiance	5.8 (3.6)	225 (27.7)	54 (6.7)	532 (65.6)
Female caregiver				
Affective	5.4 (3.7)	204 (35.7)	44 (7.7)	323 (56.6)
Anxiety	6.3 (4.3)	147 (25.7)	38 (6.7)	386 (67.6)
Pervasive development	10.1 (5.1)	335 (58.7)	91 (15.9)	145 (25.4)
Attention-deficit hyperactivity disorder	8.0 (3.2)	153 (26.8)	74 (13.0)	344 (60.2)
Oppositional defiance	6.2 (3.6)	175 (30.6)	44 (7.7)	352 (61.6)
Male caregiver				
Affective	4.2 (3.4)	56 (23.3)	17 (7.1)	167 (69.6)
Anxiety	5.0 (3.8)	41 (17.1)	12 (5.0)	187 (77.9)
Pervasive development	8.7 (5.1)	115 (47.9)	30 (12.5)	95 (39.6)
Attention-deficit hyperactivity disorder	6.8 (3.3)	42 (17.5)	17 (7.1)	181 (75.4)
Oppositional defiance	5.1 (3.5)	50 (20.8)	10 (4.2)	180 (75.0)
**ChildBehavior Checklist6–18**	**Mean (SD)**	**n (%)**	**n (%)**	**n (%)**
All caregivers				
Affective	4.9 (3.8)	191 (29.3)	114 (17.5)	346 (53.1)
Anxiety	4.1 (2.9)	216 (33.2)	101 (15.5)	334 (51.3)
Somatic	1.7 (2.2)	76 (11.7)	84 (12.9)	491 (75.4)
Attention-deficit hyperactivity disorder	8.2 (3.5)	226 (34.7)	108 (16.6)	317 (48.7)
Oppositional defiance	4.6 (2.8)	184 (28.3)	70 (10.8)	397 (61.0)
Conduct	5.2 (5.0)	168 (25.8)	85 (13.1)	398 (61.1)
Female caregivers				
Affective	5.1 (3.8)	142 (30.7)	88 (19.0)	233 (50.3)
Anxiety	4.2 (3.0)	164 (35.4)	71 (15.3)	228 (49.2)
Somatic	1.8 (2.2)	58 (12.5)	64 (13.8)	341 (73.7)
Attention-deficit hyperactivity disorder	8.6 (3.5)	180 (38.9)	77 (16.6)	206 (44.5)
Oppositional defiance	4.8 (2.9)	145 (31.3)	47 (10.2)	271 (58.5)
Conduct	5.5 (5.2)	130 (28.1)	68 (14.7)	265 (57.2)
Male caregivers				
Affective	4.3 (3.7)	49 (26.1)	26 (13.8)	113 (60.1)
Anxiety	3.7 (2.9)	52 (27.7)	30 (16.0)	106 (56.4)
Somatic	1.5 (2.2)	18 (9.6)	20 (10.6)	150 (79.8)
Attention-deficit hyperactivity disorder	7.3 (3.6)	46 (24.5)	31 (16.5)	111 (59.0)
­Oppositional defiance	4.1 (2.7)	39 (20.7)	23 (12.2)	126 (67.0)
Conduct	4.3 (4.5)	38 (20.2)	17 (9.0)	133 (70.7)
**Social Responsiveness Scale—Short Form**	**Mean (SD)**			
All caregivers	19.0 (9.9)			
Female caregiver	19.8 (10.3)			
Male caregiver	17.2 (8.6)			

Child diagnosis n represents the total number of cases for each diagnosis. Diagnoses are not mutually exclusive, participants may have more than one diagnosis. Other mental disorders include motor skills delay, anxiety disorders, specific learning problems, other syndromes, conduct/behavioural disorders, post-traumatic stress disorder, depressive disorders, tic disorder, eating disorder, obsessive compulsive disorder, mixed disorder of conduct and emotions, did not meet any diagnostic criteria and not able to make a diagnosis. Not able to make a diagnosis was assigned if clinicians were not able to diagnose due to other factors, for example, patients not attending appointments.

Approximately two-thirds of the families reported both parents living in the same household (n=701), and 22% described themselves as sole parents after separation. Stepfamilies (two parents with one being a stepparent) accounted for approximately 6% of the sample. Approximately 68% (n=988) of caregivers reported being in paid employment.

One in three caregivers (n=524) reported having received a mental health diagnosis, while approximately 53% (n=774) reported having a family history of mental illness. The total number of caregivers who completed the ASR was 1462. Approximately 39% (n=571) of adults scored in the subclinical or clinical range for at least one Diagnostic and Statistical Manual of Mental Disorders (DSM)-oriented scale, with the majority of those reporting depressive problems.

### Diagnosis

Diagnoses were obtained from the Queensland Health ieMR clinical records and were made by the Child Development Service teams. The child development service teams are multidisciplinary and include social work, speech pathology, occupational therapy, physiotherapy and developmental paediatricians. Assessments were chosen based on the presenting symptoms and hypotheses from the team. Diagnoses were based on a careful formulation after bringing together the results from the assessments. Diagnoses were provided for 814 children (table 3). Diagnoses are not mutually exclusive and up to seven diagnoses were recorded for each child. 49% of children (n=400) received one diagnosis, with the remaining 51% (n=414) receiving more than one diagnosis. Of those children receiving a diagnosis, the average number of diagnoses was 1.7 (SD=0.9). The most common diagnoses were ASD (27.8%) and ADHD (26.9%).

### CBCL and SRS

CBCL and SRS summary scores at T1 are shown in table 3. CBCL was completed by each caregiver, resulting in 811 children (preschool) and 651 (aged 6–18) surveys completed for 1084 children. 163 children had more than one CBCL survey completed by caregivers, and 212 had more than one preschool CBCL completed. In total, 248 (22.9%) children were scored within normal range on all CBCL measures by at least one caregiver. Discrepancy between caregivers reporting CBCL was observed in 78 families, that is, one caregiver scored their child in the normal range and the other parent scored their child in the clinical or subclinical range. In both the CBCL 1–5 and 6–18 measures, female caregivers’ average scores for each DSM measure were higher than the average scores, while male caregivers were consistently lower than the average score. This discrepancy between male and female caregiver scores also extended to the SRS results, with female caregivers scoring their children higher than male caregivers. This discrepancy observed between the male and female caregiver reporting is in line with previous studies.^54^ The majority of caregivers rated their children aged 1–5 in the clinical range for pervasive development, while 30% of caregivers rated children in the clinical range for ADHD in the 6–18 age group.

### Biological samples

Biological samples collected at baseline and follow-up are presented in table 4. As described previously, sample collection underwent refinement over the course of the study. As a result, DNA sample collection included blood, saliva or buccal swab. A total of 1450 participants provided DNA samples.

**Table 4:** Biological samples collected

Sample		Child	Caregiver
		**n**	**n**
Urine	Baseline	166	265
	Follow-up	39	57
Stool	Baseline	274	452
	Follow-up	32	54
DNA samples			
Blood	Baseline	124	231
	Follow-up	13	36
Saliva	Baseline	60	97
	Follow-up	1	1
Buccal swab	Baseline	445	596
	Follow-up	139	183
Total DNA	Baseline	629	924
	Follow-up	153	220

Total DNA represents totals of all DNA (blood, saliva, buccal swab) collected at baseline and follow-up.

### Summary

The IOMH cohort comprises a clinical sample of 1084 children, with a significant number experiencing neurodevelopmental issues. This cohort is relatively young, with the average age of the children being 5.6 years, and the majority were male. 77% of children were experiencing subclinical or clinical mental health symptoms. The most prevalent clinical diagnosis was ASD (27.8%) and ADHD (26.9%). Over a third of participating parents reported being diagnosed with a mental health condition. ASR DSM measures at baseline revealed caregivers were experiencing rates of mental health problems at nearly double that of the Australian 12-month prevalence rate (21.5%).^55^ 28% of families reported being sole parent families, higher than the Australian population rate of sole parents (with dependent children under 15 years) of 21%.^56^ The caregivers are highly educated, with 33% completing university study, somewhat higher than the greater Brisbane population rate of 27%.^57^ This higher level of education does not translate to higher household income, with the median household income of $50 000–99 999 per year, which aligns with the Brisbane median.^57^

### Strengths and limitations

The IOMH cohort study has a number of strengths. First, this longitudinal study tracked children with an NDD clinical diagnosis at four timepoints, allowing the trajectory of mental health conditions to be measured, along with assessing treatment efficacy. Second, a rich variety of data, including clinical, demographic, behavioural and biological, was collected, reflecting the multifaceted nature of mental disorders. This data set will provide valuable insight into the risk and protective factors of childhood mental illness. Third, where many studies focus on maternal reports on the child and parental problems,^58^ IOMH recruitment included participation of more than one caregiver, allowing a rounded picture of not only the child’s symptoms, but also the family history. Collection of DNA from the child, and one, or two, parents will provide genetic triad and dyad data which will allow more robust analysis of genetic variants. Data from multiple caregivers will also allow differences in parental perceptions to be observed.

The study was limited in that, although it was a longitudinal study, the cohort was a relatively young cohort. The study would have been strengthened by following children into adolescence and young adulthood to provide a more comprehensive overview of the trajectory of childhood mental disorders. As to be expected in a longitudinal study, dropout impacted overall participation and may have introduced a potential bias. This was further compounded by the disruptions associated with local COVID lockdown closures. Data collection concluded at the end of 2023 irrespective of which timepoint a participant had completed. All collected data were retained; however, later timepoints were discontinued where applicable, further reducing survey numbers at T3 and T4 timepoints. This also impacted the collection of biological samples at follow-up. Given this limitation, collaboration with other data sets is anticipated to generate meaningful results. Local COVID-19 restrictions and lockdowns also resulted in extensive disruption to project operation. All sample collection was suspended between March 2020 and June 2020. Lab shutdowns, staff shortages and restrictions on elective blood sample collection resulted in a backlog of both collection and lab processing. Questionnaires continued to be collected during this time; however, patient assessment numbers decreased, leading to a reduction in recruitment numbers.

Another limitation which may potentially lead to bias within the cohort is the structure of the Australian healthcare system. The Australian healthcare system is comprised of both a government-funded public system and private health insurers. Families may view the public wait times as unrealistic and opt to engage a private healthcare provider, removing themselves from this cohort and possibly creating bias in the cohort.

Finally, it was necessary to exclude children who were in the care of the Department of Child Safety due to consent limitations. As a result, children who were most vulnerable, with perhaps more severe symptoms and worse outcomes, were excluded from participation, potentially biasing the cohort.

### Future plans

Analysis in the first instance will include linear regression of baseline data, and longitudinal modelling, to investigate to what extent the course of child mental health symptoms is associated with sociodemographic and lifestyle data collected. Data linkage with the Australian Government Medicare Benefits Scheme and the Pharmaceutical Benefit Scheme will be conducted to allow modelling of treatment and demographic data in relation to behavioural outcomes.^59^ Genetic data will be used to construct PRSs for NDDs such as ASD and ADHD. Using the PRS, along with survey and clinical diagnosis data, longitudinal modelling will be used to build prediction models to investigate the factors which influence the severity and course of NDDs. In addition, data collected from the RCT will be used to investigate whether increasing the intensity of parenting programmes improves child mental health symptoms. Collection of genetic, microbiome and metabolomic data will allow for future multi-omic analysis.

### Trial registration

Embedded within the IOMH study was an RCT. The RCT is registered with the Australian New Zealand Clinical Trials Registry ANZCTR—Registration. Title: Improving outcomes in mental health for children and families: A study of Enhanced Stepping Stones Triple P. Registration number: ACTRN12618000981224.

## Data Availability

Data are available upon reasonable request.
