# Supplementary material for: Improving Outcomes in Mental Health (IOMH)—an Australian longitudinal clinical study of families with children with neurodevelopmental problems: cohort profile
Source: BMJ Open. 2025 Mar 13;15(3):e091676. doi: 10.1136/bmjopen-2024-091676 (PMC11906980; doi:10.1136/bmjopen-2024-091676)
Supplement: Supplementary file 1 [file bmjopen-2024-091676.reviewer_comments.pdf]

PEER REVIEW HISTORY

BMJ Open publishes all reviews undertaken for accepted manuscripts. Reviewers are asked to complete a checklist review form and are provided with free text boxes to elaborate on their assessment. These free text comments are reproduced below.

ARTICLE DETAILS

Title (Provisional)

Cohort Profile: Improving Outcomes in Mental Health (IOMH), An Australian Longitudinal Clinical Study of Families with Children with Neurodevelopmental Problems

Authors

Galligan, Dana Elizabeth; Payne, Leanne; Sullivan, Daniel Paul; Bhadravathi Lokeshappa, Madhura; Ziser, Laura; Nunn, Lorelle; Wallace, Leanne M; Andersen, Isabella; Howarth, Sophie; Kato, Akina; Karunanithi, Mohan; Mingin, Cassandra; O'Scanaill, Sally; Aouira , Nisreen; Paramecwari, Ayu; Sanders, Matthew R; Cobham, Vanessa; Wray, Naomi; Henders, Anjali k.; Byrne, Enda; Heussler, Honey; Middeldorp, Christel

---

VERSION 1 - REVIEW

**Reviewer**	**1**
**Name**	**Sheth, Jayesh**
**Affiliation**	**FRIGE Institute of Human Genetics, FRIGE House**
**Date**	**04-Nov-2024**
**COI**	**None**

This is a large IOMH study aimed at to improve the overall mental health assessing 1084 famlies with primary and secondary health care prooviders. They have also collected urine and stool for microbiomes and metabolomics study and also genetic study but not ahred the results of the same. This would make the ms more strong and useful scientifically for the future direction knowning the contributing factors for ASD,ADHD and which factor contribute more to the depression, anxiety and ADHD in ASD children. If author can give a briief table of this study would be great.

**Reviewer**	**2**
**Name**	**Augustine, Lilly**
**Affiliation**	**Jönköping University, School of learning and communication**

**Date**	**18-Dec-2024**
**COI**	**None**

This study focuses on the cohort profile of an Australian longitudinal clinical study of families with children with neurodevelopmental disorders (NDD). The overall aim of the Institute of Mental Health (IOMH) is to improve outcomes in mental health.

The study provides a detailed description of the cohort, which includes 1084 families and 1472 parents. This suggests that 398 families had both parents responding at the same time. However, clarity is needed regarding whether all P2 (second parent) responses are in addition to P1 (first parent) responses or if they only relate to one parent. For example, at T1, there are 203 P2 and P1 responses.

Data Collection and Follow-Up

The study describes the types of data gathered, including ratings of symptoms from parents and biological data. Although there is an intervention, it is not presented in this study. The follow-up process is explained and visually supported in Figure 2. However, the sentence on page 7, lines 3-4, could be clearer: "All caregivers were encouraged to participate, resulting in children having more than one set of survey data collected at each time-point, i.e., one set of data collected from each caregiver." figure 2 doesn't clarify if parent one always remained the same parent, or if figure 2, parent 2 indicate a second response. For example, only 22 families had both parents respond to all four cohorts, and 126 families had at least one parent respond to all four cohorts.

Intervention and Impact

Even though the Randomized Controlled Trial (RCT) is not reported, it is mentioned. Knowing the number of parents included in the intervention is crucial as it can impact outcomes at later time points. This information should be controlled for and could be included in Figure 1. Additionally, Table 1 could be incorporated into Figure 1 as an additional column detailing the questionnaire and biological samples collected.

Outcome Measures

The outcome measures include ratings of symptoms from parents and biological data. Table 2 could be better organized by having clear categories, explaining the scales used, and indicating the wave in which data was collected. This could also extend to include biological samples, making the text easier to follow.

Participant and Parent Involvement

The participant and parent involvement section should be under data collection rather than data collected. Similarly, retention and the impact of COVID-19 should be discussed in the limitations section. The impact of COVID-19 is relevant and should be included in the discussion.

Data Analysis

The data analysis is descriptive, providing central tendencies. The introduction discusses the need for biological data to understand the underlying biology of NDD, including genetics, microbiome, and metabolomic data. It is important to consider if the sample size is sufficient for analysis, especially with only 39 and 32 children at follow-up.

Summary

The summary mentions that caregivers have more mental health problems than the typical population, which aligns with previous research. However, there is no mention of the children, despite the background discussion of comorbidity. The second line in the summary on page 16 could be elaborated to include information about the children.

Strengths and Limitations

Bullet point 1: The term "clinical patients" as well as disordered should be replaced with "children with a clinical diagnosis of NDD or something similar. The study tracks these children at up to four time-points, allowing the trajectory of mental health conditions (rather than disordered) to be measured and treatment efficacy to be assessed.

Bullet point four: The increased rate of attrition due to financial restraints should be mentioned, but the financial situation itself is less relevant to the reader.

Aim of the Study

The aim of this study is to describe the cohort, not to investigate the risk factors for the persistence and severity of mental health problems in children with NDDs. This distinction should be clarified, i.e. the IOMH aim and the paper's aim.

VERSION 1 - AUTHOR RESPONSE

Reviewer: 1
Dr. Jayesh Sheth, FRIGE Institute of Human Genetics, FRIGE House
Comments to the Author:
This is a large IOMH study aimed at to improve the overall mental health assessing 1084 families with primary and secondary health care providers. They have also collected urine and stool for microbiomes and metabolomics study and also genetic study but not shared the results of the same. This would make the more strong and useful scientifically for the future direction knowing the contributing factors for ASD,ADHD and which factor contribute more to the depression, anxiety and ADHD in ASD children. If author can give a brief table of this study would be great.

1. Response:
   The manuscript is a cohort profile paper with the purpose of describing the data collected and the sample characteristics. We are able to provide summary statistics for the survey data collected, however at the time of writing the biological samples have been collected but biomarker data has not been generated due to lack of funding. We have provided tallies collected for these biological samples, in case they are of interest to other researchers. Future planned manuscripts will provide more detailed analysis of this data. Brief outlines of future genetic analysis and planned publications have been included in the Future Plans section.

Reviewer: 2
Dr. Lilly Augustine, Jönköping University
Comments to the Author:
This study focuses on the cohort profile of an Australian longitudinal clinical study of families with children with neurodevelopmental disorders (NDD). The overall aim of the Institute of Mental Health (IOMH) is to improve outcomes in mental health.

2. The study provides a detailed description of the cohort, which includes 1084 families and 1472 parents. This suggests that 398 families had both parents responding at the same time. However, clarity is needed regarding whether all P2 (second parent) responses are in addition to P1 (first parent) responses or if they only relate to one parent. For example, at T1, there are 203 P2 and P1 responses.

   Response:
   In this study a family consists of a parent labelled as P1, if a second parent participates then the second parent is labelled P2 for the entirety of the study. The P2 participants are in addition to the P1.
   Figure 2. - Upset plots are interpreted as every combination of participants and surveys timepoints they completed. To find the total number of parents who completed a T1 survey you would add across the bars in which the T1 is marked. The 203 refers to P2s who only completed a T1 survey and no further surveys. Another example, 75 P1's only completed T1 and T3.
   In this instance the upset plot was used as it illustrated that the patterns of timepoint completion in some cases was not linear. Some parents skipped surveys but later returned complete later timepoints, while others dropped out of the study after completing one timepoint.

   The caption for Figure 2 Survey completion for by Caregivers has been updated from:

‘Note. Upset plot displays the completion of surveys at each timepoint by caregivers represented by dots. P1 = Parent 1, P2 = Parent 2, Other = Caregiver who is not a parent, for example, a grandparent.’

To:
‘Caption : Note. Upset plot displays the completion of surveys at each timepoint by caregivers represented by dots. Each caregiver in a family is uniquely identified by either: P1 = Parent 1, P2 = Parent 2, Other = Caregiver who is not a parent, for example, a grandparent.’

The following was added to The Cohort Description – Participants:
‘Caregivers within each family were assigned a label to distinguish each family member (P1 = Parent 1, P2 = Parent 2, Other = Caregiver who is not a parent, for example, a grandparent). These labels remained with the participant for the entirety of the study.’

Data Collection and Follow-Up

3. The study describes the types of data gathered, including ratings of symptoms from parents and biological data. Although there is an intervention, it is not presented in this study.

   Response:
   A brief introduction has been provided for the RCT. The Authors believed that inclusion of results from the RCT is outside the scope of this manuscript and a separate manuscript is currently being drafted. This is noted in the Future Plans section.

   The RCT information in Data Collection has been expanded from:
   ‘A randomised controlled trial (RCT) was also embedded in the longitudinal study for families in which parents scored in the sub-clinical or clinical range in any of the Adult Self Report scales at T1. The RCT aimed to investigate if addressing parental mental health symptoms was beneficial to child mental health.’

   To:
   ‘A randomised controlled trial (RCT) was also embedded in the longitudinal study for families in which parents scored in the sub-clinical or clinical range in any of the Adult Self Report scales at T1. The RCT aimed to investigate if participation in an enhanced parenting program (Enhanced Stepping Stones Triple P) addressing parental mental health symptoms provided additional benefits to child mental health over and above a standard parenting program (Stepping Stones Triple P) .’

4. The follow-up process is explained and visually supported in Figure 2. However, the sentence on page 7, lines 3-4, could be clearer: "All caregivers were encouraged to participate, resulting in children having more than one set of survey data collected at each time-point, i.e., one set of data collected from each caregiver."

   Response:
   This sentence has been edited from:

‘All caregivers were encouraged to participate. This resulted in some children having more than one set of survey data collected at each timepoint i.e., one set of data collected from each caregiver.’
To:
‘All caregivers in each family were encouraged to participate, allowing each caregiver to provide information on their own symptoms and family history as well as on their child’s mental health symptoms. Hence, for some children there are two parents or carers providing information on them. ’

5. Figure 2 doesn't clarify if parent one always remained the same parent, or if figure 2, parent 2 indicate a second response. For example, only 22 families had both parents respond to all four cohorts, and 126 families had at least one parent respond to all four cohorts.

   Response:
   Each parent in the study was assigned a unique numerical identifier at T1 which was used for survey and sample identification and was retained for the duration of the study. Each family was assigned a unique family identifier to allow grouping of parents. In addition to this within a family unit a parent was identified as the P1 or P2, this status remained with the individual parent for the entire study. Rather than this being an identifier of a 1 or 2 parent family, P1/P2 was an identifier within a family unit that which remained with them throughout the study.

   The caption for Figure 2 has been amended from:
   ‘Note. Upset plot displays the completion of surveys at each timepoint by caregivers represented by dots. P1 = Parent 1, P2 = Parent 2, Other = Caregiver who is not a parent, for example, a grandparent.’
   To:
   Note. Upset plot displays the completion of surveys at each timepoint by caregivers represented by dots. Each caregiver in a family is uniquely identified by either: P1 = Parent 1, P2 = Parent 2, Other = Caregiver who is not a parent, for example, a grandparent.

   The following was added to The Cohort Description – Participants:
   ‘Caregivers within each family were assigned a label to distinguish each family member (P1 = Parent 1, P2 = Parent 2, Other = Caregiver who is not a parent, for example, a grandparent). These labels remained with the participant for the entirety of the study.’

Intervention and Impact

6. Even though the Randomized Controlled Trial (RCT) is not reported, it is mentioned. Knowing the number of parents included in the intervention is crucial as it can impact outcomes at later time points. This information should be controlled for and could be included in Figure 1.

   Response:

Randomised controlled trial families have been added to Figure 1. The authors agree that participation in a randomised controlled trial can impact outcomes for children and parents at later timepoints, however as the cohort is a clinical cohort all children regardless of their participation in the intervention will receive treatment as part of their care. This may or may not include interventions similar to the intervention provided as part of the RCT. Participants eligible to take part in the RCT were identified at T1 and the RCT intervention commenced shortly after. This allowed participants to receive the intervention prior to clinical treatment.

Outcome Measures

7. Additionally, Table 1 could be incorporated into Figure 1 as an additional column detailing the questionnaire and biological samples collected.
   And:
   The outcome measures include ratings of symptoms from parents and biological data. Table 2 could be better organized by having clear categories, explaining the scales used, and indicating the wave in which data was collected. This could also extend to include biological samples, making the text easier to follow.

   Response:
   The Table 1 has been amended to include:
   1. Table 1 Data Collection Schedule,
   2. Table 2 Questionnaire data and,
   3. Biological Samples collected has been added to the table.

   Table 2 has been removed as it is now incorporated in Table 1.

   Explanations of scales used is included in the text (Data Collected – Questionnaire, and Biological Sample Collection for associated questionnaires).

Participant and Parent Involvement

8. The participant and parent involvement section should be under data collection rather than data collected.

   Response:
   The Participant and patient involvement section has been moved to the data collection section as advised. This paragraph has been edited for context in this section.

   Participant and patient involvement section original:
   ‘Participant and patient involvement
   A parents of patients group was consulted for feedback surrounding the survey. From this feedback questions regarding positive experiences and siblings were incorporated into the survey. Feedback from participants regarding biological collections was taken into consideration and implemented when practicable. Throughout the study participants were provided regular updates through email newsletters. Research results will be shared with participants by email.’

   The Participant and patient involvement in Data Collection section now reads:

‘A parents of patients group was consulted for feedback surrounding the survey. Feedback from participants regarding biological collections was taken into consideration and implemented when practicable. Throughout the study participants were provided regular updates through email newsletters. Research results will be shared with participants by email.’

Other edits surrounding participant and patient involvement:
Data Collected – Questionnaire has been updated to include:
‘After the initial survey development, a parents/carers of patients group was consulted for feedback surrounding the survey. From this feedback questions regarding positive experiences and siblings were incorporated into the survey.’

In addition, the first sentence of Biological Sample Collection has been changed from:
‘Biological sample collection was refined and adapted over the duration of the study due to funding, COVID-19 restrictions, and participant uptake’
To:
‘Biological sample collection was refined and adapted over the duration of the study due to funding, COVID-19 restrictions, participant feedback and uptake.’

9. Similarly, retention and the impact of COVID-19 should be discussed in the limitations section. The impact of COVID-19 is relevant and should be included in the discussion.

   Response:
   The Impacts of COVID-19 paragraph has been moved to the limitations section as advised.
   The Retention paragraph however has been moved to the Data collection section. As this paragraph was describing methods rather than impacts of retention it was decided that it was better suited in the Data collection section alongside the data collection methods and timing.

Data Analysis

10. The data analysis is descriptive, providing central tendencies. The introduction discusses the need for biological data to understand the underlying biology of NDD, including genetics, microbiome, and metabolomic data. It is important to consider if the sample size is sufficient for analysis, especially with only 39 and 32 children at follow-up.

    Response:
    The authors agree with your comments. Data collection concluded in 2023 and this resulted in some families not given the opportunity to provide follow up surveys or biological samples impacting final collection tallies. This was noted in the Discussion in regard to survey data, an additional statement regarding biological data has been added:
    This also impacted the collection of biological samples at follow up. Given this limitation collaboration with other datasets is anticipated to generate meaningful results.

In addition to this, collaborations are invited in the Collaboration section.

Summary

11. The summary mentions that caregivers have more mental health problems than the typical population, which aligns with previous research. However, there is no mention of the children, despite the background discussion of comorbidity. The second line in the summary on page 16 could be elaborated to include information about the children.

    Response:
    Summary has been extended from:
    ‘The IOMH cohort comprises a clinical sample of 1084 children with a significant number experiencing neurodevelopmental issues. These children are also experiencing high rates of comorbid anxiety and depression compared to the general public child and adolescent rates of anxiety (6.9%) and depression (2.8%).’
    To:
    ‘The IOMH cohort comprises a clinical sample of 1084 children with a significant number experiencing neurodevelopmental issues. This cohort is relatively young with the average age of the children being 5.6 years, and the majority were male. Seventy-seven percent of children were experiencing sub-clinical or clinical mental health symptoms. The most prevalent clinical diagnosis was ASD (17.8%) and ADHD (17.2%).’

Strengths and Limitations

12. Bullet point 1: The term "clinical patients" as well as disordered should be replaced with "children with a clinical diagnosis of NDD or something similar. The study tracks these children at up to four time-points, allowing the trajectory of mental health conditions (rather than disordered) to be measured and treatment efficacy to be assessed.

    Response:
    This has been amended to:
    ‘Firstly, this longitudinal study tracked children with a NDD clinical diagnosis at four timepoints, allowing the trajectory of mental health conditions to be measured along with assessing treatment efficacy.’

13. Bullet point four: The increased rate of attrition due to financial restraints should be mentioned, but the financial situation itself is less relevant to the reader.

    Response:
    This sentence has been changed from:
    ‘Families with higher incomes who are able to afford private health insurance may view the public wait times unrealistic and opt to engage a private healthcare provider, removing themselves from this cohort and possibly creating bias in the cohort.’
    to
    ‘Families may view the public wait times unrealistic and opt to engage a private healthcare provider, removing themselves from this cohort and possibly creating bias in the cohort.’

14. Aim of the Study
    The aim of this study is to describe the cohort, not to investigate the risk factors for the persistence and severity of mental health problems in children with NDDs. This distinction should be clarified, i.e. the IOMH aim and the paper's aim.

    Response:
    The wording in the Abstract has been changed from:
    'The study aims to investigate risk factors for the persistence and severity of mental health problems in children with NDDs.'
    To:
    'The IOMH Cohort described in this paper aims to investigate risk factors for the persistence and severity of mental health problems in children with NDDs.'

In addition to the reviewers' comments, Table 4 has been updated to for both clarity and conciseness. As the total sample numbers represent samples at baseline and then at follow-up, it was thought that the total samples may be misconstrued for the total number of participants providing samples. As such, the total number of samples has been removed from each sample type.
